# Trajectory Planning of Autonomous Underwater Vehicles Based on Gauss Pseudospectral Method

**DOI:** 10.3390/s23042350

**Published:** 2023-02-20

**Authors:** Wenyang Gan, Lixia Su, Zhenzhong Chu

**Affiliations:** 1 Logistics Engineering College, Shanghai Maritime University, Shanghai 201306, China; 2 School of Mechanical Engineering, University of Shanghai for Science and Technology, Shanghai 200093, China

**Keywords:** autonomous underwater vehicle, trajectory planning, Gauss pseudospectral method, obstacle avoidance, cubic spline interpolation

## Abstract

This paper aims to address the obstacle avoidance problem of autonomous underwater vehicles (AUVs) in complex environments by proposing a trajectory planning method based on the Gauss pseudospectral method (GPM). According to the kinematics and dynamics constraints, and the obstacle avoidance requirement in AUV navigation, a multi-constraint trajectory planning model is established. The model takes energy consumption and sailing time as optimization objectives. The optimal control problem is transformed into a nonlinear programming problem by the GPM. The trajectory satisfying the optimization objective can be obtained by solving the problem with a sequential quadratic programming (SQP) algorithm. For the optimization of calculation parameters, the cubic spline interpolation method is proposed to generate initial value. Finally, through comparison with the linear fitting method, the rapidity of the solution of the cubic spline interpolation method is verified. The simulation results show that the cubic spline interpolation method improves the operation performance by 49.35% compared with the linear fitting method, which verifies the effectiveness of the cubic spline interpolation method in solving the optimal control problem.

## 1. Introduction

With the increasing attention to marine resources, underwater exploration and development technology have become hotspots in marine research in various countries. The autonomous underwater vehicle (AUV) has become an important tool for marine resources development due to its strong autonomy and controllability, low operational risk, economic advantages and other performance characteristics [1]. In a complex and limited marine environment, an AUV should avoid obstacles by moving forward and backward, traversing, floating and sinking, while completing tasks such as maintenance [2], underwater search [3] and detection [4]. As it is difficult for an AUV to supplement energy in the process of performing tasks, it is of great practical significance to find an optimal trajectory with low energy consumption and autonomous obstacle avoidance capability.

The trajectory planning problem is also called the trajectory optimization problem. It is essentially a nonlinear optimal control problem, which satisfies state constraints, control constraints and path constraints. According to the given starting point and destination, a trajectory is planned that satisfies the task requirements in time and space [5]. At present, most of the research results of motion trajectory planning theory originate from the fields of aircraft [6], industrial robots [7], unmanned aerial vehicles [8] and autonomous vehicles [9], while research on trajectory planning in the field of AUVs is still in the initial exploration stage. Trajectory optimization is usually solved in two steps: trajectory optimization model transformation and parameter optimization.

The main methods of trajectory optimization model transformation are the indirect and the direct methods [10]. The indirect methods solve the optimal control problem using the maximum principle. Although their calculation accuracy is high, there are some problems, such as the cumbersome derivation of the optimal solution and the difficult estimation of the covariant initial values. The process of computing problems with complex constraints is very difficult, and no relevant research has been found in the field of AUV trajectory planning. The direct methods have received wide attention. Among these methods, the pseudospectral method has been the hotspot of trajectory planning research in recent years [11]. There are three main types of traditional pseudospectral methods: the Legendre pseudospectral method (LPM), the Gauss pseudospectral method (GPM) and the Radau pseudospectral method (RPM). The differences lie in the selection and configuration of the collocation points, which affect the accuracy of the optimal solution, the convergence speed and the calculation efficiency [12]. In [13], a solution strategy based on the GPM was presented to optimize the fuel-efficient winglet retractable climb trajectory for near-space variable-wing aircraft. In [14], a multi-objective excavation trajectory optimization framework based on the RPM is proposed for the trajectory planning of the unmanned electric shovel in autonomous mining scenarios. In [15], the trajectory optimization problem is numerically solved via a hp-pseudospectral sequential convex programming method, which iteratively solves a sequence of second-order cone programming subproblems until convergence is attained. The hp-adaptive pseudospectral method combines the finite element method with the traditional pseudospectral method. It can adjust the position and the number of discrete points adaptively, and has a high solving accuracy and speed [16]. In [17], the hp-adaptive pseudospectral method was applied to the unmanned aerial vehicle trajectory planning and the minimum fuel consumption and minimum climb time obtained were optimized. However, in the field of trajectory planning of underwater vehicles, most of the existing methods do not take into account the dynamic characteristics and constraints of the controlled object in trajectory planning, which makes it difficult to track the planned trajectory and to achieve real optimization.

For parameter optimization problems, common methods include the sequential quadratic programming (SQP) algorithm, the dynamic programming algorithm, Pontragin’s method, etc. At present, the SQP algorithm is usually used to solve nonlinear programming problems [18]. Pontryagin’s method allows for control based on the comparison of the current attitude to a time-varying desired attitude, allowing for much better use of control effort and command over slew orientation [19]. In [20], where the simulation was compiled using both sinusoidal and Pontryagin trajectories, Pontryagin’s method produced an autonomous trajectory that was more optimal than the sinusoidal autonomous trajectory. The Gauss Pseudospectral Optimization Software (GPOPS), which is based on the quadratic programming algorithm, is the most widely used toolbox for the optimization of trajectory parameters [21]. In [22], the MATLAB software package Sparse Nonlinear Optimizer (SNOPT) was used to solve the nonlinear programming problem. However, in the process of parameter optimization, the existing research focuses more on the accuracy of motion trajectory solutions. Because the position and constraint conditions of an AUV are dynamic, it requires that the AUV trajectory planning must be a rolling optimization method based on time windows, and its parameter optimization must have a rapid solution speed.

With the aim of addressing the above problems, combined with the working characteristics of AUVs, a multi-constraint trajectory optimization model is constructed and solved. The main work of this paper is as follows:

(1) The motion model and the obstacle model are established, in which low energy consumption and short time are considered as the joint optimization objectives. The GPM and SQP are used to optimize the obstacle avoidance trajectory of AUVs, and the trajectory satisfying the optimization objectives and constraints is obtained.
(2) Considering the dynamic characteristics of AUVs in the complex underwater environments, the mathematical model of AUV trajectory planning is improved by introducing dynamic constraints. The navigation trajectory obtained by the solution is more in line with the actual requirements and has better traceability.
(3) When solving the trajectory planning problem based on the GPM, cubic spline interpolation is used to generate the initial values of variables. The search cost is reduced, the calculation speed is increased, the motion trajectory obtained by solving is smoother and the fitting effect is better.

The main structure of this paper is as follows: In Section 2, the trajectory planning problem of AUVs is introduced, and the motion model, the obstacle constraint model, the energy consumption and sailing time optimization model of AUVs are established. The trajectory optimization problem of AUVs is described by a mathematical model. In Section 3, the principle and process of the GPM and the SQP algorithm used to solve the trajectory planning problem are introduced. The initial value is generated by cubic spline interpolation and the optimal solution of the trajectory planning problem is obtained by iterative calculation. In Section 4, a motion trajectory satisfying the optimization objective and multiple constraints is obtained through simulation. The effectiveness of the proposed method for the trajectory planning is verified. Section 5 summarizes the research conclusions of this paper.

## 2. Problem Description

The trajectory planning problem of autonomous underwater vehicles (AUVs) in this paper can be described as follows: under the condition that the motion constraints and obstacle avoidance constraints are satisfied, finding the appropriate control amount, so that the AUV has the lowest energy consumption and the shortest sailing time when it reaches the target point. In contrast with path planning, the mathematical model of trajectory planning is generally expressed as a function related to time. Moreover, when the Gauss pseudospectral method (GPM) is used to solve the optimal control problem, the functions and equations describing the trajectory planning problem must be continuous and differentiable. Therefore, the motion constraints and obstacle constraints in the trajectory planning model need to meet the continuously differentiable conditions.

### 2.1. Motion Model of Underwater Robot

Under water, the motion of an AUV is generally 6 degrees of freedom, which can be decoupled into horizontal and vertical plane motion. This paper only discusses the horizontal motion of an AUV. An AUV’s mathematical model mainly includes the kinematics model and the dynamics model. The kinematics model mainly describes the relationship between the position, velocity, acceleration and time. The dynamic model mainly describes the motion characteristics of an AUV according to its mass and force.

If the rolling and pitching motions of the AUV are ignored, the kinematic model of the AUV in the horizontal motion plane can be expressed as follows:

(1) $$\left\{\begin{array}{l} \dot{x}=u\cdot \cos\psi -v\cdot \sin\psi ,\\ \dot{y}=u\cdot \sin\psi +v\cdot \cos\psi ,\\ \dot{\psi }=r. \end{array}\right.$$

where *x*, *y* and *ψ* represent the position and the direction angle of an AUV in a fixed coordinate system, and *u*, *v* and *r* represent the velocity and the angular velocity of an AUV in a moving coordinate system.

An AUV is mainly subject to thrust, self-gravity and buoyancy when moving underwater. The dynamic equations of an AUV in the horizontal motion plane are expressed as follows [23]:

(2) $$\left\{\begin{array}{l} m_{u}\cdot \dot{u}=m_{v}\cdot v\cdot r-d_{u}\cdot u+T_{u},\\ m_{u}\cdot \dot{v}=-m_{u}\cdot u\cdot r-d_{v}\cdot v+T_{v},\\ m_{r}\cdot \dot{r}=m_{\mathrm{uv}}\cdot u\cdot v-d_{r}\cdot r+T_{r}. \end{array}\right.$$

(3) $$\left\{\begin{array}{l} m_{u}=m-X_{\dot{u}},m_{v}=m-Y_{\dot{v}},\\ m_{r}=I_{z}-N_{\dot{r}},m_{\mathrm{uv}}=m_{u}-m_{v},\\ d_{u}=-X_{u}-X_{\left|u\right|\cdot u}\cdot \left|u\right|,\\ d_{v}=-Y_{v}-Y_{\left|v\right|\cdot v}\cdot \left|v\right|,\\ d_{r}=-N_{r}-N_{\left|r\right|\cdot r}\cdot \left|r\right|. \end{array}\right.$$

where *m* is the mass of the AUV; $I_{z}$ is the moment of inertia; ${\left(u,v,r\right)}^{T}$ is the velocity vector; *X*_(·),_ *Y*_(·)_ and *N*_(·)_ are the hydrodynamic coefficients; and $T={\left(T_{u},T_{v},T_{r}\right)}^{T}$ is the system input consisting of thrust and thrust torque.

### 2.2. Obstacle Constraint Model

Considering the complexity of entity modelling and the real-time computing ability of a computer, the underwater dangerous area and obstacle models are abstracted into several simple geometric figures in this study. As mentioned earlier, the function used to describe obstacles in trajectory planning must be continuous. Therefore, the function of approximate shape is used to define obstacles. At present, most of the mathematical models describing obstacles use the *P* criterion method. This method can conveniently represent circles, rectangles, squares, etc. [24]. Although *p* = 2 can be used to describe spherical or cylindrical obstacles, a larger *p* value should be selected in practical application to make the obstacle description more accurate. However, an excessive *p* value will increase computational cost, and the solution of complex dynamic equations may be not convergent [25]. In this paper, *p* = 2 is selected to represent the shape of obstacles with circular or elliptical envelopes.

The position relationship between the trajectory points of the AUV and the obstacle can be expressed as follows:

(4) $$h_{i}={\left(\frac{x\left(t\right)-x_{i}}{a}\right)}^{2}+{\left(\frac{y\left(t\right)-y_{i}}{b}\right)}^{2}-1,i=1,\ldots ,n$$

where (*x*(*t*), *y*(*t*)) represent the trajectory coordinates of the AUV, and (*x_i_*, *y_i_*) represent the center position of the *i*-th obstacle. The radius of the obstacle in both directions is defined by *a* and *b*. Changing *a* and *b* can adjust the size of the obstacle.

Any point (*x*, *y*) that makes *h* = 0 is on the boundary of the obstacle. When planning the obstacle avoidance trajectory of the AUV, the point (*x*, *y*) must be outside the obstacle to ensure that *h* > 0.

### 2.3. Energy Consumption and Sailing Time Optimization Model

When performing tasks, an AUV carries limited energy. To ensure the safe recovery of an AUV, energy consumption must be considered. The structural design, sailing time, sailing distance and propeller affect energy consumption, and establishing an appropriate energy consumption model is essential to achieve energy consumption optimization. The working area of an AUV is continuously changing, and the sailing speed also changes with time, so the model can be solved in the form of an integral. Another optimization objective in the trajectory planning problem is the sailing time. In numerical analysis and calculation, the position, attitude, velocity and acceleration of an AUV can be expressed as a function or equation of sailing time.

The goal of trajectory planning in this paper is to realize the joint optimization of energy consumption and sailing time, and to calculate the trajectory of an AUV reaching the destination with the lowest energy consumption and the shortest sailing time. In order to achieve the dynamic balance of the two types of objectives, weight coefficients *ω*_1_ and *ω_2_* are introduced, and the weighted coefficient method is used to construct the joint optimization objective function as follows:

(5) $$J=\omega_{1}t_{f}+\omega_{2}\int_{0}^{t_{f}}\left(T_{u}{}^{2}+T_{v}{}^{2}+T_{r}{}^{2}\right)dt$$

where *ω*_1_ *+ ω*_2_ *=* 1 and *t_f_* represent the terminal time of navigation, and $T_{u},T_{v},T_{r}$ represent the thrust and torque generated by the propeller.

To meet the navigation planning of different requirements, the weight coefficients *ω*_1_ and *ω*_2_ of the energy consumption and navigation time should be adjusted according to the actual situation. When *ω*_1_ *=* 1 or *ω*_2_ *=* 1, the trajectory planning achieves the single objective of the shortest sailing time or the lowest energy consumption, respectively.

## 3. Motion Trajectory Planning

The Gauss pseudospectral method (GPM) studied in this paper is a kind of collocation method, which uses a global interpolation polynomial to approximate the trajectory of state variables and control variables on a series of Legendre-Gauss (LG) points. Compared with the general collocation method, the GPM can obtain higher solution accuracy and faster convergence speed with fewer nodes when solving optimal control problems [26]. Trajectory planning based on the GPM mainly includes two parts: optimal control problem transformation and nonlinear programming problem solving.

### 3.1. Transformation of Optimal Control Problem

The direct method to solve the trajectory planning problem is to transform it into a nonlinear programming (NLP) problem through discretization [27]. Because the collocation points of the GPM are all distributed on the interval [−1,1], the time variable τ∈[−1,1] is introduced. The interval $t\in [t_{0},t_{f}]$ is discretized to [−1,1] and the transformation form is represented as follows:

(6) $$\tau =\frac{2t}{t_{f}-t_{0}}-\frac{t_{f}+t_{0}}{t_{f}-t_{0}}$$

*N* LG points and the initial time $\tau_{0}=-1$ are selected as the collocation points and the state variables are approximated by *N* + 1 degree Lagrange interpolation polynomials.

(7) $$x\left(\tau \right)\approx X\left(\tau \right)=\overset{N}{\underset{i=0}{\sum }}L_{i}\left(\tau \right)x\left(\tau_{i}\right)$$

where the interpolation basis function $L_{i}\left(\tau \right)\left(i=0,1,2,\cdots ,N\right)$ is defined as follows:

(8) $$L_{i}\left(\tau \right)=\overset{N}{\underset{j=0,j\neq i}{\prod }}\frac{\tau -\tau_{j}}{\tau_{i}-\tau_{j}}$$

The control variable is approximated by the *N* degree Lagrange interpolation polynomial.

(9) $$u\left(\tau \right)\approx U\left(\tau \right)=\overset{N}{\underset{i=1}{\sum }}\tilde{L_{i}}\left(\tau \right)U\left(\tau_{i}\right)$$

where the interpolation basis function $\bar{L_{i}}\left(\tau \right)\left(i=1,2,\cdots ,N\right)$ is defined as follows:

(10) $$L_{i}\left(\tau \right)=\overset{N}{\underset{j=1,j\neq i}{\prod }}\frac{\tau -\tau_{j}}{\tau_{i}-\tau_{j}}$$

Differentiating Equation (7), $\dot{x}\left(\tau_{k}\right)$ can be obtained as follows:

(11) $$\dot{x}\left(\tau_{k}\right)\approx \dot{X}\left(\tau \right)=\overset{N}{\underset{i=0}{\sum }}\dot{L_{i}}\left(\tau_{k}\right)X\left(\tau_{i}\right)=\overset{N}{\underset{i=0}{\sum }}D_{ki}X\left(\tau_{i}\right)$$

The differential of the Lagrange polynomial at the LG points can be determined by off-line calculation of the differential approximation matrix $D\in R^{N\times \left(N+1\right)}$.

The state variables on each LG point are represented as $X_{1N},X_{2N},X_{3N},X_{4N},X_{5N},X_{6N}\in R^{N}$, where *X*_1*N*_, *X*_2*N*_, *X*_3*N*_ represent the position (*x*, *y*) and heading angle *ψ* of AUV, respectively, and *X*_4*N*_, *X*_5*N*_, *X*_6*N*_ represent velocity (*u*, *v*) and angular velocity *r*, respectively. The control variables are represented as *U*_1*N*_, *U*_2*N*_ and *U*_3*N*_. The dynamic constraints of the optimal control problem are transformed into algebraic constraints through the differential approximation matrix, and the integral form of the state equation is obtained as follows:

(12) $$\left\{\begin{array}{l} DX_{1N}=\frac{tf-t0}{2}\left(X_{4N}\cos X_{3N}-X_{5N}\sin X_{3N}\right)\\ DX_{2N}=\frac{t_{f}-t_{0}}{2}\left(X_{4N}\sin X_{3N}+X_{5N}\cos X_{3N}\right)\\ DX_{3N}=\frac{t_{f}-t_{0}}{2}X_{6N}\\ DX_{4N}=\frac{t_{f}-t_{0}}{2}\left(m_{v}X_{5N}X_{6N}-d_{u}X_{4N}+U_{1N}\right)/m_{u}\\ DX_{5N}=\frac{t_{f}-t_{0}}{2}\left(-m_{u}X_{4N}X_{6N}-d_{v}X_{5N}+U_{2N}\right)/m_{u}\\ DX_{6N}=\frac{t_{f}-t_{0}}{2}\left(m_{\mathrm{uv}}X_{4N}X_{5N}-d_{r}X_{6N}+U_{3N}\right)/m_{r} \end{array}\right.$$

The terminal state $X_{f}$ is approximated by the Gauss integral in discretization.

(13) $$X_{f}=X_{0}+\frac{t_{f}-t_{0}}{2}\overset{N}{\underset{k=1}{\sum }}\omega_{k}f\left(X\left(\tau_{k}\right),U\left(\tau_{k}\right),\tau_{k},t_{0},t_{f}\right)$$

where $\tau_{k}\left(1,2,\cdots ,N\right)$ is the LG point and $\omega_{k}=\int_{-1}^{1}L_{i}\left(\tau \right)d\tau$ is the Gauss weight.

The obstacle constraint is expressed as follows:

(14) $${\left(\frac{X_{1N_{i}}-x_{i}}{a}\right)}^{2}+{\left(\frac{X_{2N_{i}}-y_{i}}{b}\right)}^{2}-1\geq 0$$

The performance index function obtained by the Gauss integral approximation is expressed as follows:

(15) $$J=\omega_{1}t_{f}+\frac{t_{f}-t_{0}}{2}\cdot \omega_{2}\cdot \overset{N}{\underset{k=1}{\sum }}\left[\omega_{k}\cdot \left(T_{u}{}^{2}+T_{v}{}^{2}+T_{r}{}^{2}\right)\right]$$

So far, the GPM has transformed the optimal control problem into an algebraically constrained nonlinear programming problem through discretization.

### 3.2. Rapid Solution Method of Trajectory

Based on the above numerical approximation method, the continuous optimal control trajectory planning problem is discretized and transformed into a nonlinear programming problem, whose solution is an approximate solution of the continuous Bolza problem. The nonlinear programming problem can be solved by SQP, which essentially transforms the nonlinear optimization problem into a series of quadratic programming sub-problems.

According to Equations (12)–(15), the transformed NLP constraint problem is described as follows:

(16) $$\begin{array}{l} \min J=\omega_{1}t_{f}+\frac{t_{f}-t_{0}}{2}\cdot \omega_{2}\cdot \overset{N}{\underset{k=1}{\sum }}\left[\omega_{k}\cdot \left(T_{u}{}^{2}+T_{v}{}^{2}+T_{r}{}^{2}\right)\right]\\ s.t.\;\left\{\begin{array}{l} DX_{1N}-\frac{tf-t0}{2}\left(X_{4N}\cos X_{3N}-X_{5N}\sin X_{3N}\right)=0\\ DX_{2N}-\frac{t_{f}-t_{0}}{2}\left(X_{4N}\sin X_{3N}+X_{5N}\cos X_{3N}\right)=0\\ DX_{3N}-\frac{t_{f}-t_{0}}{2}X_{6N}=0\\ DX_{4N}-\frac{t_{f}-t_{0}}{2}\left(m_{v}X_{5N}X_{6N}-d_{u}X_{4N}+U_{1N}\right)/m_{u}=0\\ DX_{5N}-\frac{t_{f}-t_{0}}{2}\left(-m_{u}X_{4N}X_{6N}-d_{v}X_{5N}+U_{2N}\right)/m_{u}=0\\ DX_{6N}-\frac{t_{f}-t_{0}}{2}\left(m_{\mathrm{uv}}X_{4N}X_{5N}-d_{r}X_{6N}+U_{3N}\right)/m_{r}=0\\ X_{f}-X_{0}+\frac{t_{f}-t_{0}}{2}\overset{N}{\underset{k=1}{\sum }}\omega_{k}f\left(X\left(\tau_{k}\right),U\left(\tau_{k}\right),\tau_{k},t_{0},t_{f}\right)=0\\ {\left(\frac{X_{1N_{i}}-x_{i}}{a}\right)}^{2}+{\left(\frac{X_{2N_{i}}-y_{i}}{b}\right)}^{2}-1\geq 0 \end{array}\right. \end{array}$$

Taylor expansion is used to simplify the nonlinear constraint problem into a linear function, and the following quadratic programming problem is obtained as follows:

(17) $$\begin{array}{l} \min\frac{1}{2}d_{k}^{T}B_{k}d_{k}+{\left(\nabla J\right)}^{T}d_{k}\\ s.t.\left\{\begin{array}{l} {\left(\nabla C_{e}\left(x\right)\right)}^{T}d_{k}+C_{e}\left(x\right)=0\\ {\left(\nabla C_{i}\left(x\right)\right)}^{T}d_{k}+C_{i}\left(x\right)=0 \end{array}\right. \end{array}$$

where $C_{e},C_{i}$ represent the equality constraints and inequality constraints in Equation (16), respectively.

The search direction is obtained by solving Equation (17), and the iterative equation is calculated step by step to obtain the optimal solution to the original problem.

When solving based on the GPM, the selection of the initial value is very important for the real-time or fast calculation of optimization problems. A prerequisite for real-time or fast optimization is an efficient initial value generation algorithm. Random initial values or initial values without any processing will increase the computational cost. If the calculation is performed with poor quality initial values, then the optimization problem may not converge to a feasible solution.

For discrete points between the start and target points, a simple way to obtain values is linear fitting. A straight line connects the discrete points, and the initial value is obtained by taking values with equal intervals in the interval. Because the linear fitting function is not smooth enough, the solution calculation speed is slow when the initial value is substituted into the trajectory planning problem, and the resulting navigation trajectory is more complicated. Therefore, the cubic spline interpolation method is considered to calculate the initial value of variables. The variable value [*X*_0_, *X_f_*] from the initial state to the terminal state is divided into n intervals $\left[(x_{0},x_{1}),(x_{1},x_{2}),\ldots ,(x_{n-1},x_{n})\right]$, with a total of *n* + 1 points, where $x_{0}=X_{0},x_{n}=X_{f}$. In the interval, the constructed cubic spline function is defined as follows:

(18) $$S_{i}\left(x\right)=a_{i}+b_{i}x+c_{i}x^{2}+d_{i}x^{3}i=0,\ldots ,n$$

It can be seen from Equation (18) that each interval has four unknowns, and the cubic spline equation of each interval satisfies the following conditions:

(19) $$\left\{\begin{array}{l} S(x_{i})=y_{i}\\ S_{i}(x_{i+1})=y_{i+1}\\ S_{i+1}(x_{i+1})=y_{i+1}\\ S_{i}^{\prime }(x_{i+1})=S_{i+1}^{\prime }(x_{i+1})\\ S_{i}^{\prime \prime }(x_{i+1})=S_{i+1}^{\prime \prime }(x_{i+1})\\ S^{\prime \prime }(x_{0})=S^{\prime \prime }(x_{n})=0 \end{array}\right.$$

The spline coefficients $a_{i},b_{i},c_{i},d_{i}$ are solved by Equation (19), and the cubic spline function in each interval is obtained after substituting $a_{i},b_{i},c_{i},d_{i}$ into $S_{i}(x)$. The values at the LG points are taken as the initial values for solving the trajectory planning problem. By introducing the cubic spline interpolation method, the fitted curve trajectory is smoother and more realistic, and the initial value of the interpolation calculation can improve the solution speed of the optimization problem.

## 4. Simulation Analysis

In order to verify the effectiveness of the above trajectory planning algorithm, this paper conducts an optimization simulation for the joint objective of low energy consumption and short sailing time. The starting state of the autonomous underwater vehicle (AUV) is set to $X_{0}=\left(0,0,\pi /3,0,0,0\right)$ and the target state is set to $X_{f}=\left(25,25,\pi /6,0,0,0\right)$. The constraints of state variables and control variables during sailing are expressed as follows:

(20) $$\left\{\begin{array}{l} 0\;m\leq x\left(t\right)\leq 30\;m\\ 0\;m\leq y\left(t\right)\leq 30\;m\\ -\pi \leq \psi (t)\leq \pi \\ -1\;m/s\leq u\left(t\right)\leq 1\;m/s\\ -1\;m/s\leq v\left(t\right)\leq 1\;m/s\\ -\pi /6\;\mathrm{rad}/s\leq r\left(t\right)\leq \pi /6\;\mathrm{rad}/s \end{array}\right.$$

(21) $$\left\{\begin{array}{l} -53\;N\cdot m\leq \tau_{u}\leq 74\;N\cdot m\\ -53\;N\cdot m\leq \tau_{v}\leq 74\;N\cdot m\\ -53\;N\cdot m\leq \tau_{r}\leq 74\;N\cdot m \end{array}\right.$$

The relevant parameters in the kinetic constraints are shown in Table 1.

The obstacle parameters in the navigation environment are shown in Table 2, and there are four obstacles in total.

In the above simulation environment, 50 LG points are selected to discretize the model and then an SQP algorithm is used to solve the problem.

**Figure 1:**
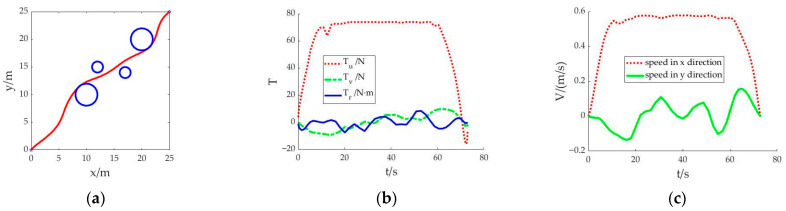
Result of each state in scenario 1: (**a**) Trajectory planning of the AUV; (**b**) Variation diagram of thrust; (**c**) Diagram of velocity change in x and y directions.

Scenario 1: The shortest sailing time is set as the objective function. The cubic spline interpolation method is used to calculate the initial value for the solution. After the simulation calculation, the navigation trajectory of the AUV and the change trend of the control variables and state variables are obtained as shown in Figure 1. The whole simulation calculation takes 16.581 seconds.

In Figure 1a, the planned trajectory can complete the navigation from the starting point to the destination and avoid collision with obstacles. Due to the requirement of the objective function of the shortest sailing time, the AUV’s navigation path tends to be smooth. The sailing time is 73.002 seconds. In Figure 1b, under the condition of meeting the requirements of the AUV’s own thrust and torque, the thrust *T_u_* in the control variable is always in the upper limit during the whole navigation process. In Figure 1c, within the limit of speed, the power generated by thrust and torque forces the AUV to sail at the upper limit of speed. Thus, the AUV can reach the destination with the shortest sailing time.

**Figure 2:**
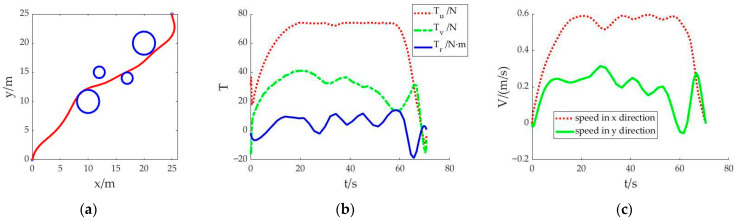
Result of each state in scenario 2: (**a**) Trajectory planning of the AUV; (**b**) Variation diagram of thrust; (**c**) Diagram of velocity change in x and y directions.

Scenario 2: Similarly to scenario 1, the shortest sailing time is still set as the objective function. However, the difference is that the initial value is generated by linear fitting that takes values at equal intervals between the start point and the end point. After substituting the initial value for calculation, the convergence solution of the optimal control problem can be obtained. The obtained trajectory is shown in Figure 2. The whole simulation calculation takes 24.763 seconds.

Figure 2a shows that the AUV can achieve obstacle avoidance navigation. The whole navigation path is generally smooth, but after bypassing the last obstacle, the AUV’s course deviates. The sailing time is 70.713 seconds. In Figure 2b, the change trend of *T_u_* in the control variables is generally smooth. Within the limits of thrust, the AUV is driven to sail with the upper limit of thrust. The values of control variables *T_v_* and *T_r_* have obvious fluctuations, which affected the speed change of the AUV. During the whole voyage, the speed fluctuates frequently, as shown in Figure 2c.

**Figure 3:**
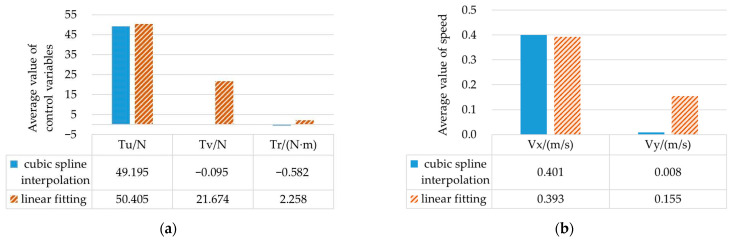
Average value of control variables and state variables in scenario 1 and scenario 2: (**a**) Average value of thrust and torque; (**b**) Average value of speed in x and y directions.

By comparing the simulation results of scenario 1 and scenario 2, it can be concluded that the initial values generated by the cubic spline interpolation method and the linear fitting method, respectively, can be used to find the convergent solution. The trajectory planned from the starting point to the target point can avoid collision with obstacles, and the control variables can be optimized within the range of variable constraints. However, the average values of control variables and the average values of the speed of the two methods are significantly different, as shown in Figure 3.

In Figure 3a, the thrust *T_u_* in the linear fitting method is 21.769 N higher than that in the cubic spline interpolation method. In Figure 3b, the change of thrust makes the speed *V_y_* in the linear fitting method 0.147 m/s higher than that in the cubic spline interpolation method. In the case of similar sailing time, the thrust and speed calculated by the cubic spline interpolation method can reduce energy consumption and optimize the control variable value. 

Under the same simulation environment, the calculation time of the initial value obtained by the cubic spline interpolation method is shorter than that of the initial value obtained by taking the values at equal intervals. The solution time of the optimal control problem is reduced from 24.763 s to 16.581 s, and the calculation speed is increased by 49.35%. The calculation results meet certain accuracy requirements, which can verify the validity and applicability of the GPM in trajectory planning applications, with the initial value obtained by the cubic spline interpolation method.

**Figure 4:**
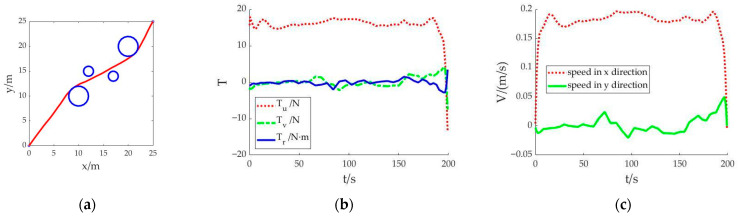
Result of each state in scenario 3: (**a**) Trajectory planning of the AUV; (**b**) Variation diagram of thrust; (**c**) Diagram of velocity change in x and y directions.

Scenario 3: Short time taken and low energy consumption are considered as the joint optimization objectives, and the cubic spline interpolation method is used to generate the initial value. The motion trajectory obtained after solving is shown in Figure 4. The whole simulation calculation takes 16.427 s.

The navigation trajectory in Figure 4a shows that the solved motion trajectory is smoother with the joint objectives of short time taken and low energy consumption than that in scenario 1 and scenario 2. The whole sailing distance is shorter. After bypassing the obstacles, the AUV sailed almost in a straight line. In Figure 4b, the control variable *T_u_* is stable at about 15 N. The sailing time is 199.305 s. It can be seen that the reduction in energy consumption is at the expense of increased sailing time.

**Figure 5:**
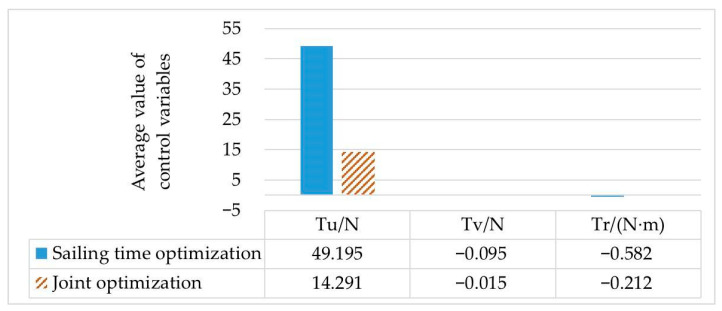
Comparison diagram of control variables in scenario 1 and scenario 3.

By comparing scenario 1 and scenario 3, it can be seen that the calculation results of the optimal control quantity are different with different set objective functions, as shown in Figure 5. When the AUV is required to reduce energy consumption, the average value of the control variable *T_u_* is only 14.291 N from the starting point to the destination, and the total distance is the shortest in these three cases. The calculation times of scenario 1 and scenario 3 are similar, which also proves that the initial value generated by the cubic spline interpolation method can improve the speed of solving the optimal control problem.

From the above simulation results, it can be concluded that these two methods of generating initial values can quickly calculate the motion trajectory and the values of the optimal control variables. Under different objective function scenarios, the obtained trajectories can meet the requirements of obstacle avoidance. In the same simulation environment, the solution speed of the cubic spline interpolation method is faster than that of the linear fitting method. When the objective function is changed and the cubic spline interpolation method is used simultaneously, the thrust distribution results are significantly different. When only focusing on the goal of sailing time, the propeller drives the AUV to sail with the upper limit of thrust to ensure that it reaches the destination in the shortest time.

## 5. Conclusions

In this paper, the problem of autonomous underwater vehicles (AUVs) motion trajectory planning based on the GPM is studied, and low energy consumption and short sailing time are considered as joint optimization objectives. At the same time, dynamic constraints are added to the model to make the planned trajectory more reasonable and more trackable. The optimal solution of the control variables can be obtained after solving the trajectory optimization problem. The planned trajectory of the AUV can meet the requirements of obstacle avoidance. The model can be solved according to different objective functions, and the value of control variables can be adjusted to meet the actual requirements of underwater navigation.

In the process of parameter optimization, the selection of the initial value affects the speed of solving the optimal control problem. The linear fitting method and the cubic spline interpolation method are proposed to calculate the initial value. The simulation results show that the calculation time of the cubic spline interpolation method is less than that of the linear fitting method under the same simulation environment. Compared with the linear fitting method, the cubic spline interpolation method reduces the global calculation amount of the trajectory optimization problem and improves the computational performance by 49.35%. Moreover, the trajectory calculated by the cubic spline interpolation method is smoother and the control variable value is more stable.

As a rapid solution for the optimal control problem, the simulation results verify the effectiveness of the initial value generated by the cubic spline interpolation method, which provides a useful reference for the real-time planning of a trajectory. In future work, in accordance with the rapidity of the solution of this model, we will embed this model into the AUV control system to verify the effectiveness of real-time planning and realize online planning.

## Figures and Tables

**Table 1:** Parameters related to dynamic constraints.

Name	Value	Name	Value
$X\dot{u}$	−30 kg	$N\dot{r}$	−30 kg∙m^2^
Xu	−70 kg/s	Nr	−50 kg∙m/s
X|u|⋅u	−100 kg/m	N|r|⋅r	−100 kg/m
$Y\dot{v}$	−80 kg	Iz	50 kg∙m^2^
Yv	−100 kg/s	m	185 kg
Y|v|⋅v	−200 kg/m		

**Table 2:** Obstacle parameters.

Obstacle Area	Center Coordinates	Radius
1	(10 m, 10 m)	2
2	(12 m, 15 m)	1
3	(17 m, 14 m)	1
4	(20 m, 20 m)	2

## Data Availability

Not applicable.

## Abbreviations

The following abbreviations are used in this manuscript:

AUV	Autonomous Underwater Vehicle
GPM	Gauss Pseudospectral Method
SQP	Sequential Quadratic Programming
LPM	Legendre Pseudospectral Method
RPM	Radau Pseudospectral Method
GPOPS	Gauss Pseudospectral Optimization Software
SNOPT	Sparse Nonlinear Optimizer
LG points	Legendre-Gauss points
NLP	Nonlinear Programming

## Nomenclatures

The following nomenclatures are used in this manuscript:

*x*	Coordinate position in x direction
*y*	Coordinate position in y direction
*ψ*	Direction angle of the AUV
*u*	Velocity in x direction
*v*	Velocity in y direction
*r*	Angular velocity of the AUV
*m*	Mass of the AUV
*I_z_*	Moment of inertia
*X_(·)_*	Hydrodynamic coefficient
*Y_(·)_*	Hydrodynamic coefficient
*N_(·)_*	Hydrodynamic coefficient
*T_u_*	Thrust generated by the propeller
*T_v_*	Thrust generated by the propeller
*T_r_*	Thrust torque generated by the propeller
*t_f_*	Terminal time of navigation
*h_i_*	Obstacle function
*a*	Radius of the obstacle
*b*	Radius of the obstacle
*J*	Objective function
*ω*_1_	Weight coefficient
*ω_2_*	Weight coefficient
*τ*	Time variable
*L_i_(τ)*	Interpolation basis function
*D*	Differential approximation matrix
*x(τ)*	State variable function
*u(τ*)	Control variable function
*X_f_*	Terminal state
*τ_k_*	LG points
*ω_k_*	Gauss weight
*C_e_*	Equality constraints
*C_i_*	Inequality constraints
*a_i_*	Spline coefficient
*b_i_*	Spline coefficient
*c_i_*	Spline coefficient
*d_i_*	Spline coefficient
*S_i_(x)*	Cubic spline function
